# The association between stigmatizing attitudes towards depression and help seeking attitudes in college students

**DOI:** 10.1371/journal.pone.0263622

**Published:** 2022-02-18

**Authors:** Virgínia Conceição, Inês Rothes, Ricardo Gusmão

**Affiliations:** 1 EPIUnit—Institute of Public Health of the University of Porto (ISPUP), Porto, Portugal; 2 Laboratory for Integrative and Translational Research in Population Health (ITR), Porto, Portugal; 3 Faculty of Psychology and Education Science, University of Porto, Porto, Portugal; 4 Center for Psychology at University of Porto, Porto, Portugal; 5 Department of Public Health and Forensic Sciences, and Medical Education, Faculty of Medicine, University of Porto, Porto, Portugal; Gachon University Gil Medical Center, REPUBLIC OF KOREA

## Abstract

Depression stigma has been considered a significant barrier to treatment and rehabilitation. This study aimed to understand the effects of gender, previous mental health care, and symptomatology on depression stigma and analyze the impact of depression stigma on help-seeking attitudes. A total of 969 students with a mean age of 18.87 (SD = 1.49) were included in this study and completed the Depression Stigma Scale, the Attitude Toward Seeking Professional Psychological Help, the Patient Health Questionnaire-4 questionnaire, and a socio-demographic questionnaire. We analyzed data using SPSS 24.0, with a 95% confidence interval. Participants came from all University schools, and 64.6% were women. Personal stigma and help-seeking attitudes were affected by gender (β_(male)_ = 5.65, CI = 4.07, 7.25) and previous access to mental healthcare services (β_(previous help)_ = -4.35, CI = -5.89, -2.82). Perceived depression stigma was affected gender (β_(male)_ = -2.67, CI = -5.00, -0.34) and symptomatology (β_(no symptomatology)_ = -3.29, CI = -6.09, -0.49). Personal (r = -0.42, p<0.01) and perceived (r = 0.10, p<0.01) depression stigma correlated with help-seeking attitudes, but we detected no direct symptomatology effect on help-seeking attitudes. Personal depression stigma significantly affected help-seeking attitudes (β = -0.15, CI = -0.17, -0.12). Promoting literacy may decrease personal depression stigma and increase professional help-seeking attitudes and behaviors.

## Introduction

Mental and behavioral disorders are estimated to account for 12% of the global burden of disease [1]. The number of people diagnosed with depressive disorders amounts to 4.4% of the world population, and depression ranks as the first contributor to non-fatal health burden, with 7.5% of all Years Lived with Disability (YLD) [2].

College years are a critical developmental phase that coincides with emerging adulthood [3], conceptualized as ’late adolescence’ [4]. Entering a college or university mainly presents a high-risk transition due to situational changes and upturns in life stressors, including academic and interpersonal difficulties [5]. Mental illness negatively impacts academic performance, increasing school attrition odds [6]. First-year college students have been identified as a risk group for developing depression, with reports of higher prevalence among university students than non-students for the same age group [7].

People affected by depressive disorders face peer rejection [8], social and institutional stigmatizing responses similar to psychosis or chronic mental illness [9], with non-recognition of mental disorders as general health illness both by health professionals and society in general [10], healthcare access constraints [11], and increased difficulties in finding and maintaining a paid job [12].

Thus, reducing the stigma affecting people who have a mental illness is one of the World Health Organization Mental Health Action Plan 2013–2020 main objectives [13]. Identifying predictors of stigma is an important starting point to address stigmatizing attitudes [14].

Much of the recent research on depression stigma has focused on two types of social stigma: personal stigma and perceived stigma [15]. Personal stigma refers to one’s beliefs about depression, and perceived stigma refers to one’s beliefs about the attitudes of others [14]. Research on these two types of stigma has reported lower personal stigma levels than perceived stigma [16].

In the last decade, there has been a growing interest in mental health stigma interventions and research in Portugal. A European study centered on depression stigma and attitudes towards professional help in the general adult population [17] identified Portugal as the second country (in four) with the highest level of personal and perceived depression stigma, with 31.2% of the participants agreeing with personal stigmatizing affirmations, and 53.8% agreeing with perceived stigma affirmations, measured with the Depression Stigma Scale [18]. In the Attitudes Towards Seeking Professional Help scale, Portugal ranked in the first place for openness to professional help; however, the value of professional service was not as good, with only 48.2% agreeability. Lower schooling levels, being a male, and not having previous contact with people with mental illness were associated with increased stigma scores [19].

Attention has been made to mental health stigma in health students considering health professionals may act as literacy promoters [20]. In the past years, research about stigma in health students in Portugal has demonstrated the need for stigma reduction intervention in this group of students [21, 22].

Help-seeking has been a particular problem for first-year university students. Most of the WHO World Mental Health International College Student Initiative report [23] participants reported they would hesitate to seek help if needed. This report identified attitudinal barriers, such as stigma, as the most critical constraint in help-seeking.

Despite the increasing number of studies addressing mental health stigma and help-seeking [24–26], most studies neglect to integrate the symptomatology of participants in the analysis.

Also, sampling in young adults has been a problem; usually, participants are health students or professionals [27], resulting in a lack of studies contemplating all study areas of the universities.

Lastly, the effects of previous mental healthcare utilization have not been well explored and can be helpful in the study of the relationship between stigma and help-seeking [24].

With this study, we aim to (1) examine whether socio-demographic characteristics are related to personal and perceived stigma in students, (2) to understand the effects of the depressive and anxiety symptoms of depression stigma, and (3) examine whether stigmatizing attitudes towards depression is associated with help-seeking behavior in students.

## Methods

### Sample

In February 2019, we contacted all 4493 first-year students from the 14 schools of the University of Porto through institutional email using the SurveyMonkey platform and invited them to participate in the study. A reminder email was sent a week after the first email. At the end of the questionnaire, a list of available healthcare responses was available, so students could seek help if they felt it was needed.

To be included in the study, participants needed to have answered all items in the questionnaire and be under 25 years old, the recognized late adolescent age limit [4].

### Measures

We asked participants to answer a socio-demographic questionnaire assessing gender, age, place of birth, university course, the Attitudes Toward Seeking Professional Psychological Help (ATSPPH) [28], the Patient Health Questionnaire-4 (PHQ-4) [29], and the Depression Stigma Scale (DSS) [18].

Previous access to mental health care was assessed with the "yes" or "no" question "did you ever go to a health care professional due to symptoms related to your mental health". The "yes" answer was coded as "with previous mental health care", and the answer "no" was coded as "no previous mental health care".

The ATSPPH is a ten-item four-point Likert scale, with scores varying from 0 to 30. The scale ranges from "disagree" (score 0) to "agree" (score 3) and comprises two subscales: the first one evaluates the openness to seeking treatment for emotional problems (items 1, 3, 5, 6, and 7), and the second evaluates the value and need on seeking treatment (items 2, 4, 8, 9 and 10, reversed before summed). The maximum score in each subscale is 15, and higher scores indicate better attitudes toward seeking treatment. ATSPPH is the most used scale in mental health help-seeking attitudes, showing excellent psychometric properties in its original form. One of the authors was responsible for translating and adapting the ATSPPH to the Portuguese population in the OSPI program—Optimizing suicide prevention programs and their implementation in Europe—and showing a Cronbach’s alpha of 0.80 [17, 30, 31].

PHQ-4 is a 4-item questionnaire with two items measuring depressive symptoms and two items for anxiety symptoms. Its score varies from 0 to 12; scores 0–4 represent absent symptomatology, 5–8 mild symptomatology, and 9–12 severe symptomatology [32, 33]. The PHQ-4 used in the study resulted from the extraction of the corresponding items from the Portuguese version of the PHQ-9 (Cronbach’s alpha = 0.87) [34] and GAD-7 (Cronbach’s alpha = 0.88) [35].

DSS is an 18 five-point Likert scale, with the first nine items measuring personal stigma (one’s own beliefs about depression) and the last nine items measuring perceived stigma (one’s beliefs about the attitudes of others). We converted the total score of both scales as a percentage according to the original score calculation [18], and the higher the percentage, the higher the stigma. The scale has been translated and validated to the Portuguese population using the OSPI program data, obtaining a Cronbach’s alpha of 0.71 in the personal subscale and 0.75 in the perceived subscale [17, 30, 31].

### Ethical considerations

The study complies with the relevant national and institutional ethical standards on human experimentation and the Helsinki Declaration of 1975, as revised in 2008. The Institute of Public Health of the University of Porto ethics committee approved the research with the ID reference CE18096. All participants signed an informed consent digital form following the Helsinki and Oviedo Conventions.

### Data analysis

We performed the analysis using SPSS 24 at a 95% confidence level.

We used percentages to describe participation rates, gender distribution, moving away from home, living situation, presence of mental illness in the family, and previous access to mental healthcare. We used counts to describe participant distribution per faculty. Symptomatology stemming from the PHQ-4 scores resulted in three possible severity categories (absent, mild, and severe), and we presented the distribution per category in percentages.

First, we calculated descriptive statistics: frequencies, means, and standard deviations. Then, we analyzed variance to examine gender differences, experience with previous healthcare, symptoms, depression stigma, and help-seeking attitudes using One-Way ANOVA with Tukey HSD’s multiple comparisons test among groups. To test for the possible interaction effect between gender and previous mental healthcare on depression stigma and help-seeking attitudes, we performed a Two-Way ANOVA.

In normally distributed continuous variables (personal and perceived depression stigma and help-seeking attitudes), we performed T-tests to study differences between the gender, symptomatology groups, and previous access to mental healthcare.

We calculated effect sizes using the formula for Cohen’s d test = $\frac{M2‐M1}{{\mathrm{SD}}_{\mathrm{pooled}}}$, where $\sqrt{\frac{{SD}_{1}^{2}+{SD}_{2}^{2}}{2}}$ and the Partial eta square using the formula η_p_^2^ = $\frac{{\mathrm{SS}}_{\mathrm{effect}}}{{{\mathrm{SS}}_{\mathrm{effect}}+\mathrm{SS}}_{\mathrm{error}}}$.

We carried out a Pearson correlation to examine associations between personal depression stigma and perceived depression stigma and depression stigma and attitudes toward help-seeking.

Lastly, we executed hierarchical linear regressions to statistically control variables to see whether adding variables improves a model’s ability to predict the Depression Stigma and the Help-seeking attitudes scores. Variables included in the models were gender, previous contact with mental illness, mental illness in the family, and symptomatology group.

## Results

### Participants and general results

Of the 4493 students invited to participate in this study, a total of 1046 (23.3%) accepted to participate. Considering the age inclusion criteria, we included 969 participants (21.5%) in the study, all with no missing data in the questionnaire.

Respondents came from all 14 schools: 197 from Engineering, 164 from Humanities, 111 from Sciences, 82 from Biomedical Sciences, 80 from Economics, 77 from Law, 68 from Psychology and Educational Sciences, 49 from Arts, 44 from Pharmaceutical Sciences, 39 from Medicine, 22 from Nutrition, 16 from Architecture, 13 from Sports, and 7 from Dental Medicine.

About two-thirds of the students were women (64.6%, 626), and 35.4% (343) were men, with a mean age of 18.87 (SD = 1.49) ranging between 16 and 25. Approximately 43.9% of the students were away from home, and most of those (46.1%) were living with roommates.

Most participants had never previously sought mental health help care (59.6%), and 35.4% had a family member with a mental illness diagnosis. Anxiety was the main reason (46.4%) for those who had previously sought mental healthcare, followed by depression (25%).

In the PHQ-4, participants showed a mean of 5.65 (SD = 3.35), and most of the participants had no symptomatology (51.8%). However, 25.5% showed mild symptomatology and 22.7% severe symptomatology. Only 47.3% of the participants with severe symptomatology had previously sought professional help.

Pearson correlation between Personal and Perceived Depression stigma subscales was not significant: r = 0.003, p = 0.94.

### Personal depression stigma

Personal depression stigma means were very similar between schools; however, differences were statistically different: F_(956,13)_ = 2.84, *p*<0.01 (η_p_^2^ = 0.03). Post-hoc Tukey HSD’s multiple comparisons revealed that only the differences between Arts, Psychology and Educational Sciences and Engineering and Economics were significant. Arts students presented a mean of 18.59 (SD = 9.13) and Psychology and Educational Sciences students a mean of 19.61 (SD = 9.11), significantly lower mean scores than Engineering (M = 26.61, SD = 13.57) and Economics (M = 26.56, SD = 13.07) students more information can be found in S1 Table (Personal and perceived depression stigma mean scores per school) and S2 Table (Personal and perceived depression stigma Tukey HSD’s post-hoc significant differences between schools).

Women had a lower personal stigma level than males. Participants with no previous access to mental health care obtained means of personal depression stigma statistically higher (M = 28.00, SD = 13.22) than those with previous access to mental health care (M = 26.11, SD11.20), t_(968)_ = -6.08, p<0.001 (*d =* 0.41). These differences turned out to be only significant among women: t_(625)_ = -5.98, p<0.001 (*d =* 0.52), detailed information can be found in S3 Table (Personal depression stigma means differences according to gender, help-seeking, and symptomatology groups) of the supporting information. There was a significant interaction between gender and previous mental care (F_(968,1)_ = 4.85, p<0.01, η_p_^2^ = 0.05).

There was also a significant effect of symptomatology on personal depression stigma, and the mild symptoms groups presented the higher depression stigma score (see S3 Table—Personal depression stigma means differences according to gender, help-seeking, and symptomatology groups, of the supporting information). Tukey HSD’s post-hoc analysis revealed that only the mild symptomatology group differed from the other severity symptoms groups: p<0.01 between absent and mild symptoms and p<0.001 between mild and severe. When comparing depression stigma score differences among previous mental health care groups, we verify significance in those with previous mental health care; however, there was no significant interaction between symptomatology and previous mental health care (F_(968,2)_ = 0.92, p = 0.40).

Participants with a family member with a mental illness diagnosis had a mean personal stigma score of 22.32 (SD = 11.26), a significantly lower result (t_(966)_ = −2.24, p<0.05, *d* = 0.32) than those with no family member with mental illness (M = 24.17, SD = 12.78).

The hierarchical multiple regression (Table 1) revealed that at Stage one, gender contributed significantly to the regression model, F_(966,1)_ = 48.62, p<0.001, and accounted for 4.7% of the variation in Personal Depression Stigma. Introducing the previous care group variable explained an additional 2.9% variation, and this change in R^2^ was significant, F_(966,1)_ = 30.60, p <0.001. Adding family mental illness and symptoms group did not produce an R^2^ significant change. The most important predictor of personal stigma was gender which uniquely explained 4.8% of the variation.

**Table 1:** Effects of gender, previous mental care experience, family mental illness, and symptomatology group on personal depression stigma.

	β	95% CI	t	p
	**Model 1**			
Women	Ref.			
Men	**5.65**	**4.07, 7.25**	**48.61**	**<0.001**
	**Model 2**			
Women	Ref.			
Men	**5.16**	**3.58, 6.73**	**41.18**	**<0.001**
No previous mental care	Ref.			
With previous mental care	**-4.35**	**-5.89, -2.82**	**30.88**	**<0.001**
	**Model 3**			
Women	Ref.			
Men	**5.12**	**3.54, 6.69**	**40.40**	**<0.001**
No previous mental care	Ref.			
With previous mental care	**-4.20**	**-5.74, -2.65**	**20.20**	**<0.001**
Family with mental illness—No	Ref.			
Family with mental illness—Yes	-1.22	-2.80, 0.36	2.30	0.13
	**Model 4**			
Women	Ref.			
Men	**5.11**	**3.54, 6.68**	**40.74**	**<0.001**
No previous mental care	Ref.			
With previous mental care	**-4.12**	**-5.66, -2.59**	**27.43**	**<0.001**
Family with mental illness—No	Ref.			
Family with mental illness—Yes	-1.16	-2.73, 0.41	2.09	0.15
No symptoms	1.02	-0.86, 2.91	1.14	0.27
Mild symptoms	**3.65**	**1.50, 5.79**	**11.04**	**<0.01**
Severe symptoms	Ref.			

β = beta regression coefficients, Ref. = Reference category

* Model 1 = gender; Model 2: Model 1 plus mental care experience; Model 3: Model 2 plus family mental illness; Model 4: Model 3 plus symptomatology group.

Significant results are in bold.

### Perceived depression stigma

Perceived depression mean scores were similar between schools, even though statistically different: F_(956,13)_ = 2.04, *p*<0.05 (η_p_^2^ = 0.02). Post-hoc Tukey HSD’s multiple comparisons revealed that this significance was due to the differences between the School of Sports (M = 43.60, SD = 13.72), which was significantly lower than Sciences (M = 60.98, SD = 17.71), Biomedical Sciences (M = 62.80, SD = 17.56), Psychology and Educational Sciences (M = 61.84, SD = 17.91), Humanities (M = 64.78, SD = 16.11), Economics (M = 64.06, SD = 18.83) and Nutrition (M = 65.15, SD = 18.42) (see supporting information, S1 Table—Personal and perceived depression stigma mean scores per school, and S2 Table—Personal and perceived depression stigma Tukey HSD’s post-hoc significant differences between schools).

Gender and previous mental health care differences were statistically significant in the total scores of the perceived depression stigma. Women obtained a total mean score of 62.68 (SD = 18.48), and in Men M = 59.79 (SD = 16.17) (t_(968)_ = -2.43, p<0.05, *d* = 0.21); participants with previous mental health care obtained the highest mean scores (M = 62.26, SD = 18.24), compared participants with no previous mental health care (M = 60.58, SD = 17.34), t_(968)_ = 2.32, p<0.05, *d* = 0.12 (see S4 Table—Perceived depression stigma means differences according to gender, help-seeking, and symptomatology groups, of supporting information). However, the interaction between gender and the mental health care experience was not significant (F_(968,1)_ = 0.01, p = 0.94).

The severe symptoms group presented the highest perceived stigma score (see S4 Table—Perceived depression stigma means differences according to gender, help-seeking, and symptomatology groups, of the supporting information). Tukey HSD’s post-hoc analysis revealed a significant difference between the two groups: no symptoms and severe symptoms (p<0.05). The interaction between the symptoms and mental health care experience was not significant (F_(968,2)_ = 0.49, p = 0.62).

We obtained non-significant (t_(968)_ = 1.77, p = 0.08) lower scores of perceived stigma in participants with no family member with mental illness (M = 60.91, SD = 17.31) compared with those with a family member bearing a mental disorder diagnosis (M = 63.01, SD = 18.49).

Since there were no differences between participants with and without mental illness in the family, we did not include this variable in the hierarchical linear regression.

As can be observed in Table 2, gender and symptomatology scores had significant effect on perceived depression stigma. Despite the significant effects verified, the variance explained in each model is very small (0.6% in Model 1, 0,8% in Model 2, and 1.1% in model 3). Only Model 1 and Model 3 produced a R^2^ significant change: Model 1 F_(967,1)_ = 6.41, p <0.05; Model 2 F_(967,1)_ = 3.01, p = 0.08; Model 3 F_(967,1)_ = 4.27, p <0.05.

**Table 2:** Effects of gender, previous mental care experience, family mental illness, and symptomatology group on perceived depression stigma.

	β	95% CI	t	p
	**Model 1**			
Women	Ref.			
Men	**-3.05**	**-5.38, -0.72**	**6.59**	**<0.05**
	**Model 2**			
Women	Ref.			
Men	**-2.76**	**-5.09, -0.42**	**5.36**	**<0.05**
No previous mental care	Ref.			
With previous mental	**2.47**	**0.19, 4.74**	**4.50**	**<0.05**
	**Model 3**			
Women	Ref.			
Men	**-2.67**	**-5.00, -0.34**	**5.04**	**<0.05**
No previous mental care	Ref.			
With previous mental	2.26	-0.02, 4.54	3.78	0.05
No symptoms	**-3.29**	**-6.09, -0.49**	**5.32**	**<0.05**
Mild symptoms	**-3.28**	**-6.48, -0.08**	**4.04**	**<0.05**
Severe symptoms	Ref.			

β = beta regression coefficients, Ref. = Reference category

* Model 1 = gender; Model 2: Model 1 plus mental care experience; Model 3: Model 2 plus symptomatology effect. Significant results are in bold.

The most relevant predictor of perceived stigma was gender which explained merely 0.6% of the variation.

### Help-seeking attitudes

Help-seeking attitudes presented a mean of 18.62 (SD = 4.76). Women showed significantly higher means (19.20, SD = 4.90) than men (17.56, SD = 4.32) (t_(966)_ = -5.20, p<0.001, *d* = 0.57). Participants with previous mental health care presented higher scores on help-seeking attitudes (20.00, SD = 4.54) than those with no previous mental health care (17.69, SD = 4.69, t_(966)_ = 7.66, p<0.001, *d* = 0.43). Differences according to symptomatology were not significant: No symptoms M = 18.77, SD = 4.93; Mild symptoms M = 18.15, SD = 4.17; and Severe symptoms M = 18.79, SD = 4.96 with an ANOVA test of F_(966,2)_ = 1.61, p = 0.20. We did not find a significant interaction between the global score of the help-seeking attitudes scale and gender, previous mental health care experience, or symptomatology group.

Participants with a family member with mental illness presented higher help-seeking scores (M = 19.22, SD = 4.80) than those with no mental illness in the family (M = 18.28, SD = 4.80), t_(966)_ = 2.95, p<0.01, *d* = 0.22.

Personal depression stigma was moderately correlated with help-seeking attitudes (r = -0.42, p<0.01). The perceived stigma showed a weaker Pearson correlation with help-seeking attitudes (r = 0.10, p<0.01).

The hierarchical multiple regression (Table 3) revealed that at Stage one, gender contributed significantly to the regression model, F_(966,1)_ = 27.62, p<0.001, and accounted for 2.8% of the variation in the general score of the help-seeking attitudes scale. Introducing the previous care variable explained an additional 4.9% variation, and this change in R^2^ was significant, F_(966,1)_ = 50.80, p <0.001. Adding family mental illness did not produce an R^2^ significant change. Personal depression stigma explained an additional 13.1% of the variance with a significant change in R^2^ (F_(966,1)_ = 159.93, p <0.001), and perceived stigma explained an additional 0.01% of the variance. Model 5 total variance explained was 21.6%.

**Table 3:** Effects of gender, previous mental health care, family mental illness, personal depression stigma, and perceived depression stigma on help-seeking attitudes.

	β	95% CI	t	p
	**Model 1**			
Women	Ref.			
Men	**-1.64**	-**2.26, -1.02**	**5.20**	**<0.001**
	**Model 2**			
Women	Ref.			
Men	**-1.39**	**-1.20, -0.79**	**4.96**	**<0.001**
No previous mental care	Ref.			
With previous mental	**2.17**	**1.58, 2.76**	**-7.18**	**<0.001**
	**Model 3**			
Women	Ref.			
Men	**-1.14**	**-1.99, -0.78**	**4.47**	**<0.001**
No previous mental care	Ref.			
With previous mental	**2.08**	**1.48, 2.68**	**-6.84**	**<0.001**
Family with mental illness–No	Ref.			
Family with mental illness–Yes	0.58	-0.02, 1.19	-1.87	0.06
	**Model 4**			
Women	Ref.			
Men	**-0.64**	**-1.21, -0.07**	**2.19**	**<0.05**
No previous mental care	Ref.			
With previous mental	**1.47**	**0.91, 1,67**	**-5.15**	**<0.001**
Family with mental illness–No	Ref.			
Family with mental illness–Yes	0.40	-0.16, 0.97	-1.41	0.15
Personal Depression Stigma	**-0.15**	**-0.17, -0.12**	**-12.64**	**<0.001**
	**Model 5**			
Women	Ref.			
Men	**-0.59**	**-1.16, -0.01**	**1.99**	**<0.05**
No previous mental care	Ref.			
With previous mental	**1.44**	**0.88, 1.99**	**-5.02**	**<0.001**
Family with mental illness–No	Ref.			
Family with mental illness–Yes	0.37	-0.19, 0.93	-1.293	0.20
Personal Depression Stigma	**-0.15**	**-0.17, -0.12**	**-12.76**	**<0.001**
Perceived Depression Stigma	**0.02**	**0.01, 0.04**	**2.53**	**<0.05**

β = beta regression coefficients, Ref. = Reference category

* Model 1 = gender; Model 2: Model 1 plus previous mental care; Model 3: Model 2 plus family mental illness; Model 4: Model 3 plus Personal Depression Stigma; Model 5: Model 4 plus Perceived Depression Stigma.

Significant results are in bold.

### Openness to seeking treatment for emotional problems

The mean for openness to seeking treatment was 8.82 (SD = 2.95), and the mean was higher among women (8.98, SD = 3.10) than men (8.53, SD = 2.64) with a significant difference: t_(966)_ = -2.67, p<0.05, *d* = 0.18. Participants with previous mental health care experience presented higher mean scores (9.71, SD = 2.92) than participants with no previous experience with mental care (8.21, SD = 2.82), and the differences were statistically significant: t_(966)_ = 7.95, p<0.001, *d* = 0.45.

Having a family member with mental illness beneficiated the openness to seeking treatment as the participants in this group obtained a higher mean (9.19, SD = 2.88) than those with no mental illness in the family (8.61, SD = 2.68), t_(966)_ = 2.92, p<0.01, *d* = 0.19. There were no differences according to symptomatology (F_(966,2)_ = 1.29, p = 0.28).

Personal depression stigma showed a moderate correlation with openness to seeking treatment (r = -0.30, p<0.01), and the correlation with perceived stigma was not significant.

The hierarchical multiple regression revealed that gender contributed significantly to the regression model at model one, F_(966,1)_ = 5.30, p<0.05, but accounted only for 0.05% of the variation. Previous care explained an additional 5.8% variation, and this change in R^2^ was significant, F_(966,1)_ = 32.87, p <0.001. Adding the family mental illness group did not produce an R^2^ significant change (F_(966,1)_ = 3.67, p = 0.06). Personal depression stigma explained an additional 12.7% of the variance with a significant change in R^2^ (F_(966,1)_ = 65.81, p <0.001). Introducing perceived stigma in the model did not result in an R^2^ significant change: F_(966,1)_ = 2.07, p = 1.51. In this final model, the total variance explained was 12.4%, however, only having previous mental health care (in comparison with not having previous mental care: β = 1.15, CI = 0.79, 1.52) and personal stigma (β = -0.06, CI = -0.08, -0.05) had a significant beta regression coefficient (see S5 Table—Effects of gender, previous help group, family mental illness, personal depression stigma and perceived depression stigma on Openness to seeking treatment for emotional problems, of the supporting information).

### Value and need of seeking treatment

Participants showed a mean of 9.81 (SD = 2.56) in the value and need of seeking treatment. Woman presented significant higher means (10.23, SD = 2.50) than men (9.03, SD = 2.49), t_(966)_ = -7.01, p<0.001. Participants who have previously sought help obtained higher mean scores (10.29, SD = 2.38) than participants with no previous care (9.47, SD = 2.63), with differences statistically significant: t_(966)_ = 4.97, p<0.001. Participants with mental illness in the family presented higher mean scores (10.03, SD = 2.48) than participants with no family member with mental illness (9.67, SD = 2.59), and these differences were statistically significant: t_(966)_ = 2.10, p<0.05.

Symptomatology did not impact mean scores for value and need of seeking treatment (F_(966,2)_ = 1.83, p = 0.16).

Personal depression stigma showed a moderate correlation with openness to seeking treatment (r = -0.45, p<0.001), and the correlation with perceived also showed a significant correlation, yet weaker (r = 0.10, p<0.01).

Gender accounted for 5% of the variation as the first variable included in the hierarchical multiple regression, with a significant contribution for the R^2^ change: F_(966,1)_ = 51.37, p<0.001. The previous care variable explained an additional 1.8% variation, and the change produced in R^2^ was also significant, F_(966,1)_ = 17.79, p <0.001. The family mental illness group did not change the R^2^ (F_(966,1)_ = 1.58, p = 0.21. Personal depression stigma explained an additional 15.3% of the variance with a significant change in R^2^ (F_(966,1)_ = 189.38, p <0.001), and perceived stigma explained an additional lower amount of variance (0.1%) yet significant in the R^2^ change: F_(966,1)_ = 8.94, p<0.01. Model 5 explained a total variance of 22.9%. The previous mental help care variable and family with mental illness group did not have a significant beta regression coefficient (see S6 Table - Effects of gender, previous help group, family mental illness, personal depression stigma and perceived depression stigma on Value and need of seeking treatment, of the supporting information).

## Discussion

We contacted students to participate in this study via their institutional email addresses. Since they do not frequently access their institutional email address, response rates were relatively low. Thus, the response rate was lower than the mean in similar studies in the general population [36]. Another explanation may be the recent increase in online surveys. Nevertheless, there was a comparable response rate within each school, except for Economics, with a much higher response rate than the mean, and Sports and Architecture, with a lower than 10% response rate.

The gender distribution is comparable to the population: in the 2018–2019 school year, the University of Porto’s records show that 59% of the students were women and 41% men.

The Portuguese population’s personal depression stigma mean score was 32.1% in the Coppens and colleagues study, conducted under the OSPI program [17]. Our sample presented lower stigma scores, which may be due to our sample’s much lower mean age. It is established in the literature an association between depression stigma and older age [17, 19].

Concordant to previous studies [16], women presented lower depression stigma levels than men, which may be due to the masculine set of social norms [37] and expected behavior associated with being men [38].

Both gender and t previous mental health care group significantly affected personal depression stigma, unlike having a family member with mental illness. Symptomatology was a significant predictor for mild symptoms compared with severe symptomatology.

The observed results in perceived stigma agree with previous studies, where the effects of the previous contact with depression are more evident for personal stigma than perceived stigma [14, 39].

Depressive symptoms have a mitigating effect on personal stigma for those who have previously sought help, which may be due to personal stigma mental health interventions’ reduction effects. However, previous mental health care experience did not have the same stigma reduction effect in men and women, and future research could explore these differences more thoroughly.

Participants with milder symptoms showed more personal stigma and lower perceived stigma than those with severe symptomatology, resulting from a process of self-stigmatization and self-denial of a possible need for help, especially in the case of personal stigma. This particularity may be an essential element to consider when addressing personal stigma.

Past help-seeking had a more significant effect on the openness to seek treatment than on the value and need of seeking treatment, which may be interesting to research further. Previous help-care experiences are an essential tool for literacy and help-seeking behaviors promotion.

Symptomatology did not impact help-seeking attitudes, suggesting the importance of mental health literacy promotion to improve the recognized value of health care when needed. Stigma reduction might be one of college students’ primary depression help-seeking promotion strategies, considering the information available about help-seeking barriers and underlying mechanisms [40, 41].

Demonstration of a more substantial correlation of depression personal stigma with help-seeking attitudes than gender or previous contact with mental healthcare services confirms the importance of stigma interventions in promoting help-seeking behavior [11, 42]. Personal stigma concerning depression was the variable with the most significant effect on help-seeking attitudes, in line with the strong effect of attitudinal barriers in help-seeking [23].

One of the study’s main limitations is the sample’s representativeness. The high rate of previous mental healthcare service utilization suggests the possibility of selection bias. However, the prevalence of severe symptomatology identified in our sample (22.7%) is similar to the 20.3% 12-month prevalence identified in the WHO World Mental Health Survey [43]. The second limitation is the lack of data on essential elements of stigma, namely the time and duration of mental health access care. Further studies should consider these elements to understand better the effects of previous mental healthcare usage on stigma and help-seeking attitudes.

Despite the possible and inevitable sample bias in online studies, our sample compares well to the University of Porto population, both in distribution by the school and distribution by gender, strengthening the generalizability of the results.

Due to our sample’s low age variance, we only considered first-year students below 25 years old–age was not included as a study variable. Future research directed to understanding the relationship between age and depression stigma will address this gap.

Future research should also study the gender imbalance and previous mental health care between University Schools.

In conclusion, intensifying mental health promotion and easing access to mental healthcare will increase the probability of successful early intervention on college students’ depression and anxiety disorders. More research will be needed to understand the different effects of mental health services on stigma reduction among men and women.

These findings also reinforce the importance of stigma reduction interventions as a tool to promote help-seeking behaviors. Therefore, future research on the actual effect of stigma reduction on healthcare use is essential.

## Supporting information

S1 Table Personal and perceived depression stigma mean scores per school. (DOCX)

S2 Table Personal and perceived depression stigma Tukey HSD’s post-hoc significant differences between schools. (DOCX)

S3 Table Personal depression stigma means differences according to gender, help-seeking, and symptomatology groups. (DOCX)

S4 Table Perceived depression stigma means differences according to gender, help-seeking, and symptomatology groups. (DOCX)

S5 Table Effects of gender, previous help group, family mental illness, personal depression stigma and perceived depression stigma on openness to seeking treatment for emotional problems. (DOCX)

S6 Table Effects of gender, previous help group, family mental illness, personal depression stigma and perceived depression stigma on value and need of seeking treatment. (DOCX)
