# Supplementary material for: The association between stigmatizing attitudes towards depression and help seeking attitudes in college students
Source: PLoS One. 2022 Feb 18;17(2):e0263622. doi: 10.1371/journal.pone.0263622 (PMC8856567; doi:10.1371/journal.pone.0263622)
Supplement: S1 Table — (DOCX) [file pone.0263622.s001.docx]

Table S1: Personal and perceived depression stigma mean scores per school

|  |  | **n** | **Personal Depression Stigma**  **M (SD)** | **Perceived Depression Stigma**  **M (SD)** |
| --- | --- | --- | --- | --- |
| Engineering | | 197 | 26.61 (13.57) | 59.75 (26.95) |
| Humanities | | 164 | 24.07 (12.06) | 64.78 (16.11) |
| Sciences | | 111 | 23.12 (12.01) | 60.98 (17.71) |
| Biomedical Sciences | | 82 | 21.81 (10.03) | 62.80 (17.56) |
| Economics | | 80 | 26.56 (13.07) | 64.06 (18.83) |
| Law | | 77 | 22.72 (15.90) | 60.35 (20.81) |
| Psychology and Educational Sciences | | 68 | 19.61 (9.11) | 61.84 (17.91) |
| Arts | | 49 | 18.59 (9.13) | 60.43 (17.94) |
| Pharmaceutical Sciences | | 44 | 24.05 (12.23) | 59.78 (17.63) |
| Medicine | | 39 | 22.22 (9.32) | 60.68 (13.31) |
| Nutrition | | 22 | 21.59 (11.78) | 65.15 (18.42) |
| Architecture | | 16 | 26.21 (10.92) | 62.67 (13.72) |
| Sports | | 13 | 26.07 (10.67) | 43.60 (13.72) |
| Dental Medicine | | 7 | 23.02 (12.39) | 54.76 (16.64) |
