# Supplementary material for: The association between stigmatizing attitudes towards depression and help seeking attitudes in college students
Source: PLoS One. 2022 Feb 18;17(2):e0263622. doi: 10.1371/journal.pone.0263622 (PMC8856567; doi:10.1371/journal.pone.0263622)
Supplement: S2 Table — (DOCX) [file pone.0263622.s002.docx]

Table S2: Personal and perceived depression stigma Tukey HSD's post-hoc significant differences between schools.

|  | Schools | | M | SE | p | CI |
| --- | --- | --- | --- | --- | --- | --- |
| Personal Depression Stigma | Arts | Engineering | -8.01 | 1.95 | <0.01 | -14.58, -1.45 |
|  |  | Economics | -7.97 | 2.21 | <0.05 | -15.43, -0.51 |
|  | Psychology and Educational Sciences | Engineering | -6.99 | 1.72 | <0.01 | -12.78, -1.22 |
|  |  | Economics | -6.95 | 2.02 | <0.05 | -13.74, -0.17 |
| Perceived Depression Stigma | Sports | Sciences | -17.40 | 5.16 | <0.05 | -34.75, -0.04 |
|  |  | Biomedical Sciences | -19.21 | 5.26 | <0.05 | -36.89, -1.54 |
|  |  | Psychology and Educational Sciences | -18.26 | 5.33 | <0.05 | -36.17, -0.33 |
|  |  | Humanities | -21.20 | 5.07 | <0.01 | -38.26, -4.14 |
|  |  | Economics | -20.47 | 5.27 | <0.01 | -38.18, 2.77 |
|  |  | Nutrition | -21.56 | 6.16 | <0.05 | -42.27, -0.84 |
