# Supplementary material for: The association between stigmatizing attitudes towards depression and help seeking attitudes in college students
Source: PLoS One. 2022 Feb 18;17(2):e0263622. doi: 10.1371/journal.pone.0263622 (PMC8856567; doi:10.1371/journal.pone.0263622)
Supplement: S3 Table — (DOCX) [file pone.0263622.s003.docx]

Table S3: Personal depression stigma means differences according to gender, help-seeking, and symptomatology groups.

|  | Total | No previous mental care n=576 | With previous mental care  n=393 | T-test_(df)_ | Cohen’s *d* |
| --- | --- | --- | --- | --- | --- |
| Mean (SD) | 23.76 (12.37) | 25.72 (12.65) | 20.88 (11.53) | **t_(968)_=-6.08, p<0.001** | *d=*0.41 |
| Men: M (SD)  n=343 | 27.37 (12.90) | 28.00 (13.22)  n=230 | 26.11 (12.20)  n=113 | t_(341)_=-1.31, p=0.19 | *d=*0.14 |
| Women: M (SD)  n=626 | 21.79 (11.63) | 24.20 (12.08)  n=348 | 18.78 (10.92)  n=278 | **t_(625)_= -5.98**, **p<0.001** | *d=*0.52 |
| t-test_(df)_ | **t_(968)_=6.88, p<0.001** | **t_(577)_= 3.56**, **p<0.001** | **t_(392)_= 6.05**, **p<0.001** |  | |
| Cohen’s *d* | *d=*0.55 | *d=*0.30 | *d=*0.65 |  | |
| Absent symptoms  (n=502) | 23.51 (11.89) | 25.11 (12.11)  n=315 | 19.97 (9.98)  n=187 | **t_(500)_= -4.92**, **p<0.001** | *d*=0.51 |
| Mild symptoms  (n=247) | 25.91 (13.19) | 27.06 (13.44)  n=145 | 23.95 (12.77)  n=102 | t_(245)_= -1.81, p=0.07 | *d*=0.19 |
| Severe symptoms  (n=220) | 21.72 (12.20) | 24.68 (13.19)  n=116 | 18.36 (10.31)  n=104 | **t_(218)_= -3.93**, **p<0.001** | *d*=0.54 |
| ANOVA_(df)_ | **F_(966,2)_=6.86, p<0.01** | F_(575,2)_=1.51, p=0.22 | **F_(388,2)_=4.20, p<0.01** |  | |
| η_p_^2^ | η_p_^2^=0.12 | η_p_^2^=0.02 | η_p_^2^0.16 |  | |

M=mean, SD=standard deviation; df=degrees of freedom. Significant results are in bold.
