# Supplementary material for: The association between stigmatizing attitudes towards depression and help seeking attitudes in college students
Source: PLoS One. 2022 Feb 18;17(2):e0263622. doi: 10.1371/journal.pone.0263622 (PMC8856567; doi:10.1371/journal.pone.0263622)
Supplement: S4 Table — (DOCX) [file pone.0263622.s004.docx]

Table S4: Perceived depression stigma means differences according to gender, help-seeking, and symptomatology groups.

|  | Total | No previous mental care n=576 | With previous mental care  n=393 | T-test_(df)_ | Cohen’s *d* |
| --- | --- | --- | --- | --- | --- |
| Mean (SD) | 61.66 (17.60) | 60.58 (17.34) | 62.26 (18.24) | **t_(968)_=2.32, p<0.05** | *d=*0.12 |
| Men: M (SD)  n=343 | 59.79 (16.17) | 58.92 (16.06) n=230 | 61.57 (16.03) n=113 | t_(341)_=1.42, p=0.15 | *d*=0.16 |
| Women: M (SD)  n=626 | 62.68 (18.48) | 61.67 (18.07) n=348 | 63.94 (18.94) n=278 | t_(625)_= 1.54, p=0.13 | *d*=0.15 |
| t-test_(df)_ | **t_(968)_=-2.43, p<0.05** | t_(577)_= -1.87, p=0.06 | t_(392)_= -1.69, p=0.24 |  | |
| Cohen’s *d* | *d*=0.21 | *d*=0.09 | *d*=0.07 |  | |
| Absent symptoms  (n=502) | 60.89 (16.80) | 60.27 (16.50) n=315 | 61.91 (17.28) n=187 | t_(500)_= 1.06, p=0.29 | *d=*0.10 |
| Mild symptoms  (n=247) | 60.99 (18.31) | 59.96 (17.90) n=145 | 62.58 (18.91) n=102 | t_(245)_= 1.10, p=0.27 | *d*=0.09 |
| Severe symptoms  (n=220) | 64.52 (18.87) | 62.40 (18.71) n=116 | 66.90 (18.86) n=104 | t_(218)_= 1.77, p=0.08 | *d*=0.11 |
| ANOVA_(df)_ | **F_(966,2)_= 3.53, p<0.05** | F_(575,2)_= 0.78, p=0.46 | F_(388,2)_= 2.68, p=0.07 |  | |
| η_p_^2^ | η_p_^2^=0.04 | η_p_^2^0.001 | η_p_^2^0.01 |  | |

M=mean, SD=standard deviation; df=degrees of freedom. Significant results are in bold.
