# Supplementary material for: The association between stigmatizing attitudes towards depression and help seeking attitudes in college students
Source: PLoS One. 2022 Feb 18;17(2):e0263622. doi: 10.1371/journal.pone.0263622 (PMC8856567; doi:10.1371/journal.pone.0263622)
Supplement: S5 Table — (DOCX) [file pone.0263622.s005.docx]

Table S5: Effects of gender, previous help group, family mental illness, personal depression stigma and perceived depression stigma on Openness to seeking treatment for emotional problems

|  | **β** | **95% CI** | **t** | **p** |
| --- | --- | --- | --- | --- |
|  | **Model 1** |  |  |  |
| Women | Ref. |  |  |  |
| Men | **-0.45** | **-0.84, -0.06** | **2.30** | **<0.05** |
|  | **Model 2** |  |  |  |
| Women | Ref. |  |  |  |
| Men | -0.28 | -0.07, 0.10 | 1.48 | 0.14 |
| No previous mental care | Ref. |  |  |  |
| With previous mental | **1.47** | **1.10, 1.84** | **-7.75** | **<0.001** |
|  | **Model 3** |  |  |  |
| Women | Ref. |  |  |  |
| Men | -0.27 | -0.65, 0.11 | 1.40 | 0.16 |
| No previous mental care | Ref. |  |  |  |
| With previous mental | **1.42** | **1.05, 1.79** | **-7.46** | **<0.001** |
| Family with mental illness - No | Ref. |  |  |  |
| Family with mental illness - Yes | 0.37 | -0.01, 0.75 | -1.92 | 0.06 |
|  | **Model 4** |  |  |  |
| Women | Ref. |  |  |  |
| Men | 0.04 | -0.34, 0.41 | -0.21 | 0.83 |
| No previous mental care | Ref. |  |  |  |
| With previous mental | **1.17** | **0.80, 1.53** | **-6.23** | **<0.001** |
| Family with mental illness – No | Ref. |  |  |  |
| Family with mental illness – Yes | 0.30 | -0.07, 0.67 | -1.58 | 0.11 |
| Personal Depression Stigma | **-0.06** | **-0.08, -0.05** | **-8.11** | **<0.001** |
|  | **Model 5** |  |  |  |
| Women | Ref. |  |  |  |
| Men | -0.62 | -0.21, 0.44 | -0.32 | 0.75 |
| No previous mental care | Ref. |  |  |  |
| With previous mental | **1.15** | **0.79, 1.52** | **-6.15** | **<0.001** |
| Family with mental illness - No | Ref. |  |  |  |
| Family with mental illness - Yes | 0.29 | -0.08, 0.65 | -1.52 | 0.13 |
| Personal Depression Stigma | **-0.06** | **-0.08, -0.05** | **-8.16** | **<0.001** |
| Perceived Depression Stigma | 0.01 | -0.01, 0.02 | 1.44 | 0.15 |

β=beta regression coefficients, Ref.=Reference category

* Model 1= gender; Model 2: Model 1 plus previous mental care; Model 3: Model 2 plus family mental illness; Model 4: Model 3 plus Personal Depression Stigma; Model 5: Model 4 plus Perceived Depression Stigma.

Significant results are in bold.
