# Supplementary material for: The association between stigmatizing attitudes towards depression and help seeking attitudes in college students
Source: PLoS One. 2022 Feb 18;17(2):e0263622. doi: 10.1371/journal.pone.0263622 (PMC8856567; doi:10.1371/journal.pone.0263622)
Supplement: S6 Table — (DOCX) [file pone.0263622.s006.docx]

Table S6: Effects of gender, previous help group, family mental illness, personal depression stigma and perceived depression stigma on Value and need of seeking treatment

|  | **β** | **95% CI** | **t** | **p** |
| --- | --- | --- | --- | --- |
|  | **Model 1** |  |  |  |
| Women | Ref. |  |  |  |
| Men | **-1.20** | **-1.53, -0.87** | **7.17** | **<0.001** |
|  | **Model 2** |  |  |  |
| Women | Ref. |  |  |  |
| Men | **-1.12** | **-1.45, -0.79** | **6.70** | **<0.001** |
| No previous mental care | Ref. |  |  |  |
| With previous mental | **0.67** | **0.37, 1.00** | **-4.03** | **<0.001** |
|  | **Model 3** |  |  |  |
| Women | Ref. |  |  |  |
| Men | **-1.11** | **-1.44, -0.79** | **6.65** | **<0.001** |
| No previous mental care | Ref. |  |  |  |
| With previous mental | **0.66** | **0.34, 0.99** | **-4.02** | **<0.001** |
| Family with mental illness - No | Ref. |  |  |  |
| Family with mental illness - Yes | 0.21 | -0.12, 0.54 | -1.26 | 0.21 |
|  | **Model 4** |  |  |  |
| Women | Ref. |  |  |  |
| Men | **-0.68** | **-0.99, -0.38** | **4.37** | **<0.001** |
| No previous mental care | Ref. |  |  |  |
| With previous mental | **0.31** | **0.01, 0.61** | **-2.02** | **<0.05** |
| Family with mental illness – No | Ref. |  |  |  |
| Family with mental illness – Yes | 0.11 | -0.19, 0.41 | -0.70 | 0.48 |
| Personal Depression Stigma | **-0.08** | **-0.10, -0.07** | **-13.76** | **<0.001** |
|  | **Model 5** |  |  |  |
| Women | Ref. |  |  |  |
| Men | **-0.65** | **-0.95, -0.34** | **4.14** | **<0.001** |
| No previous mental care | Ref. |  |  |  |
| With previous mental | 0.28 | -0.01, 0.58 | -1.87 | 0.06 |
| Family with mental illness - No | Ref. |  |  |  |
| Family with mental illness - Yes | 0.09 | -0.21, 0.37 | -0.56 | 0.57 |
| Personal Depression Stigma | **-0.09** | **-0.10, -0.07** | **-13.91** | **<0.001** |
| Perceived Depression Stigma | **0.01** | **0.01, 0.02** | **2.99** | **<0.01** |

β=beta regression coefficients, Ref.=Reference category

* Model 1= gender; Model 2: Model 1 plus previous mental care; Model 3: Model 2 plus family mental illness; Model 4: Model 3 plus Personal Depression Stigma; Model 5: Model 4 plus Perceived Depression Stigma.

Significant results are in bold.
